# Clinical effect of diode laser on peri-implant tissues during non-surgical peri-implant mucositis therapy: Randomized controlled clinical study

**DOI:** 10.4317/medoral.56424

**Published:** 2020-01-01

**Authors:** Rebeca Sánchez-Martos, Andrea Samman, Kheira Bouazza-Juanes, José-María Díaz-Fernández, Santiago Arias-Herrera

**Affiliations:** 1 Universidad Europea de Valencia. Faculty of Health Sciences. Department of Dentistry

## Abstract

**Background:**

The aim of this study is to evaluate the response to the non-surgical treatment of peri-implant mucositis using the diode laser as an adjuvant therapy in patients with implant-supported restorations, in terms of clinical variables, with respect to those patients in whom conventional non-surgical therapy is used.

**Material and Methods:**

Randomized controlled clinical trial with simple blind 3 months follow-up. Two groups of patients were established, the non-surgical mechanical debridement of the affected implants was performed in the control group (n = 34) and the diode laser therapy was also performed in the test group (n = 34). The implant was considered the study subject; the variables considered were plaque index, bleeding on probing depth, depth of probing and recession of the peri-implant mucosa. The t-Student test was used to establish the intergroup statistical differences and the analysis of variance (ANOVA) was used to measures intragroup differences over time.

**Results:**

In the revaluation at 6 weeks, we observed statistically significant differences (*p*<0.05) between the variables of plaque index and depth of probing between both groups. The test group obtained an average of 0.248 ± 0.3155 in plaque index and 0.833 ± 0.374mm in the depth of probing compared to the results obtained in the control group that was 0.558 ± 0.526 and 1,137 ± 0.222mm respectively. In the 3-month reevaluation, was also obtained great statistical significance between both groups for bleeding on probing (*p*<0.001), with values of 0.568 ± 0.282 for the control group and 0.480 ± 0.336 for the test group.

**Conclusions:**

The use of diode laser as an adjunctive therapy to the conventional treatment of peri-implant mucositis showed promising results, being more effective reducing the inflammation of the peri-implant tissue, positioning itself as a valuable tool for the treatment of peri-implant pathologies.

** Key words:**Peri-implant diseases, peri-implant mucositis, laser therapy, diode laser, biostimulation.

## Introduction

Since the 1990s, implantology has established as a new surgical discipline of dentistry (1). Nowadays treatment with dental implants in patients with total or partial edentulism is considered a predicTable surgical-prosthodontic procedure, however, is not exempt from mechanical and biological complications (2).

Biological complications include peri-implant pathologies, defined as the inflammatory processes that take place in the tissues surrounding an implant (3). Two entities are distinguished: peri-implant mucositis and periimplantitis. According to the latest definition provided by the “Consensus report from 2017 World Workshop on Periodontology”, peri-implant mucositis is defined as an inflammatory process, which develops with edema and bleeding at probing at 30 seconds, induced by dental biofilm with absence of loss of bone after bone remodeling (1,4).

Peri-implant pathologies have an increasing prevalence. In Spain, the last review carried out by Rodrigo D *et al.* in 2018 showed a prevalence of 51% for peri-implant pathologies, with a mucositis rate of 27% (5). Internationally, the most recent review carried out by Derks & Tomasi in 2015 showed a prevalence of 65% for peri-implant pathologies with 43% of patients affected by mucositis (6).

Due to the high prevalence of these pathologies, especially peri-implant mucositis, there are numerous treatment strategies. The most common is a non-surgical approach, by mechanical debridement of the affected implants with plastic curettes (7). However, this conventional treatment has limitations in the resolution of peri-implant mucositis (8,9). To overcome these limitations, different adjuvant therapies have emerged over the years, diode laser therapy being introduced very recently. Recent studies have observed that diode laser acts on two fronts, against biofilm, the main etiological factor involved in peri-implant mucositis, through bacterial decontamination and also in the regeneration of peri-implant tissues through the process of cell biostimulation (10,11).

Therefore, the hypothesis of this study considers that diode laser can provide additional clinical benefits to the conventional non-surgical therapy of peri-implant mucositis.

The main objective of this study is to evaluate the response to non-surgical treatment of peri-implant mucositis through the use of diode laser as an adjunctive therapy in patients with implant-supported rehabilitation, in terms of clinical variables, respect to patients where conventional therapy is used.

## Material and Methods

-Study design 

A randomized controlled, single blind, prospective 3-month clinical study was carried out. The design of this study was performed using the CONSORT Declaration 2010 as a guide for conducting randomized clinical trials of parallel groups (12).

- Participants

In this study, 68 patients with peri-implant mucositis who attended the Master of Advanced Oral Implantology of the European University of Valencia during the months of January to May 2019 were evaluated. The study protocol was supervised and approved by the Ethics Committee of the Research of the European University and by the Scientific Committee of the San Carlos Clinical Hospital in Madrid. All patients received an informed consent the day of the baseline visit that specified the objective of the study, the possible benefits and risks derived from the intervention, as well as their free participation in the study. All consents were given to the patients at least 24 hours before the treatment and were explained in detail by the researcher (RS).

The following inclusion criteria were considered: Patients over 18 years of age, periodontally healthy with unitary screwed implant-supported prostheses with peri-implant mucositis were included. According to the latest World Workshop on Periodontology of 2017, those implants that presented edema and bleeding on probing at 30 seconds with absence of bone loss were diagnosed with mucositis. The absence of bone loss was verified by comparing a parallel radiograph performed in the delivery of the prosthesis and a new one made at the baseline visit.

Patients with cemented unit prostheses or multiple prostheses were excluded. In the same way patients with immunological systemic diseases, who took chronic non-steroidal anti-inflammatory drugs or who had received antibiotics in the 6 months prior to the start of the study were excluded. Patients who had received non-surgical peri-implant treatment in the previous 6 months or surgery in the previous 12 months and patients irradiated in the head and neck area were also excluded.

- Interventions

Patients who accomplish the inclusion criteria and voluntarily agreed to participate in the study (n=68) were divided into two groups. The control group (n=34) received conventional non-surgical treatment of peri-implant mucositis and the test group (n=34) received in addition diode laser therapy.

Conventional non-surgical treatment

**Figure 1:**
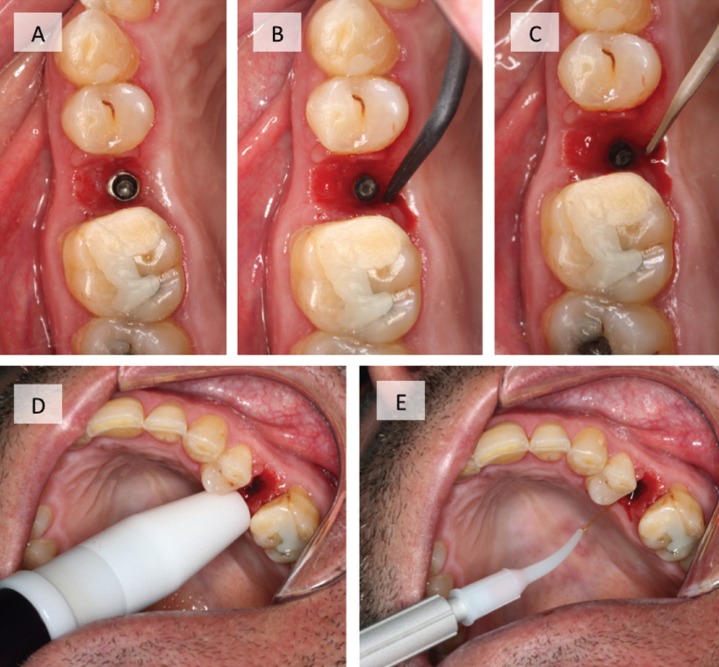
Images of the clinical protocol. Conventional non-surgical treatment of peri-implant mucositis (A, B, C): A) Removal of the crown to expose the peri-implant sulcus, B) Mechanical debridement with plastic curette, C) Mechanical debridement with plastic ultrasound tip. Diode laser therapy (D, E): D) Application of the diffuser tip, E) Application of the 300 μ optical fiber tip.

All patients (n = 68) received conventional non-surgical treatment following the protocol proposed by Renvert *et al.* in 2008 (7). The implant crown was removed, mechanical debridement was performed with plastic curettes (Implant Deplaquers®, KerrHawe SA, Bioggio, Switzerland) and with PH1 plastic ultrasound tip (Acteon Satelec®, Acteon Medical-Dental Iberian SAU, Barcelona, Spain). The peri-implant sulcus was irrigated with 0.12% chlorhexidine + 0.05% cetylpyridinium chloride (Perio-Aid Treatment®, Dentaid, Barcelona, Spain) (Fig. 1).

Diode laser therapy

Patients in the test group (n = 34) received, in addition to conventional non-surgical treatment, Fox® diode laser therapy (A.R.C. Laser GmbH, Nürnberg, Germany) distributed by Sweden & Martina (Mediterranea SL, Padua, Italy). It was used with a wavelength of 810 nm, power of 1 watt in pulsed mode and for 30 seconds per surface. First, the diffuser tip of 1 cm in diameter was applied and then the 300μ tip was used in the peri-implant sulcus parallel to the implant axis for 30 seconds (Fig. 1).

-Results

Once the informed consent was signed and received information about the objective of the study, the medical and sociodemographic variables of the sample were collected. Including: Age, gender, medical history, allergies, toxic habits, marital status, level of studies and type of profession

A preliminary calibration trial was performed for the clinical variables, in which 10 patients with peri-implant mucositis, not related with the study, were involved performing a complete periodontal examination in a duplicate manner within 2 weeks. The inter-class correlation coefficient of agreement of 0.91 was obtained.

One investigator (RS) recorded the plaque index (PI), bleeding on probing (BoP), probing pocket depth (PPD), and recession of the peri-implant mucosa (REC) at 4 locations per implant (mesio-vestibular, vestibular, disto-vestibular and palatine) using periodontal probe (North Caroline Probe, Hu-Friedy, Leinmen, Germany).

The primary outcomes were PI BoP that were transformed from categorical to quantitative variables, the rest of clinical variables and also sociodemographic and medical variables were considered as secondary outcomes.

- Sample size

The sample size was calculated in order to detect a BoP reduction (considered as a primary outcome variable) of 20% with a standard deviation (SD) of 0.96 assuming an alpha risk of 0.05 with a 95% of power; the resulting Figure was a desirable sample of 28 implants per group (13). Taking into account possible drop-outs, the total calculation of patients in the study was based on an additional 20%. Therefore, a sample size of 34 implants per group is established.

- Randomization, allocation concealment, implementation and masking mechanism

The principal investigator (SA) was in charge of randomizing the groups, using a randomized system based on stratified blocks depending on whether the patients were smokers or not. The allocation concealment was carried out through the use of sealed opaque envelopes, which were opened by a researcher (RS) during the non-surgical therapy of peri-implant mucositis. The interventions assigned to each group were performed by a calibrated and trained examiner, not blind to the group assignment (RS). The principal investigator (SA) performed the statistical analysis of the data and remained blinded after the assignment of the interventions

- Statistical methods

The implant was considered as the unit of analysis. The homogeneity of the study groups was established using the t-Student test for the age variable and the χ2 test for the rest of the socio-demographic and medical variables. All clinical variables were expressed in means with standard deviation (SD). For the intragroup differences the ANOVA test was used and also for the intergroup differences the t-Student test was used. Data were analyzed (Statistical Package for Social Sciences for MAC, SPSS Inc., Chicago, IL, USA) and the level of statistical significance was set at 5% (*p* <0.05) for all analysis.

## Results

**Figure 2:**
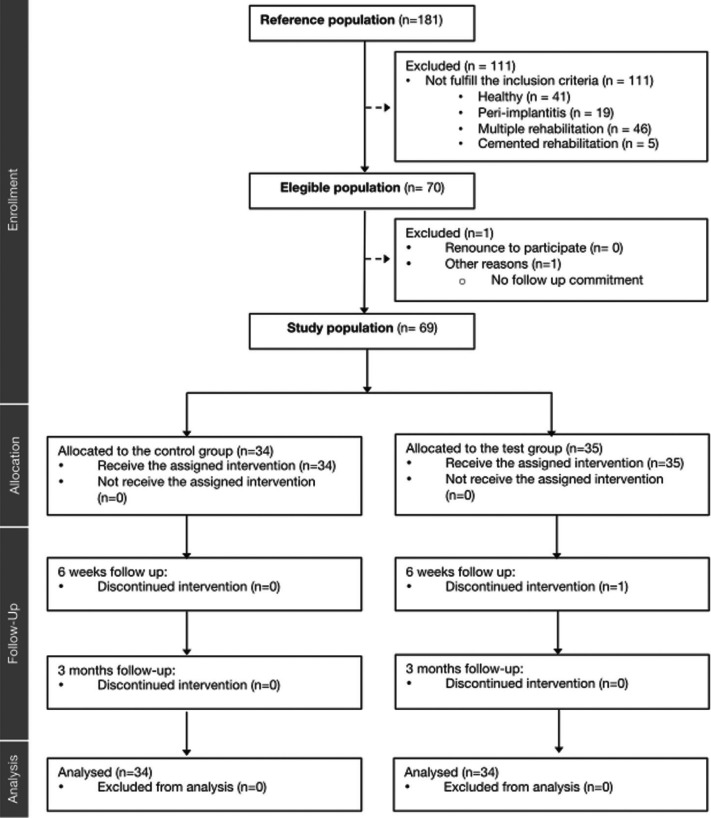
Flow-chart of the study, with number of patients.

Out of the 181-screened implants, 68 were included in the study. The implants belonged to a total of 68 patients, with a mean age of 57±11.39 years. One hundred and twelve implants were excluded because they did not fulfill the inclusion criteria, one patient refused to participate in the study and there was a loss in the test group during the 6-week follow-up phase (Fig. 2). All implants included in the study were conical implants, with internal connection and unitary screwed prostheses. Most of the implants were 3i (n= 62) (BIOMET 3i T3®, Zimmer Biomet, Indiana, USA) and the rest were Straumann implants (n=6) (Straumann BL®, Straumann GmbH, Freiburg, Germany). The implants were in function for an average time of 3,057 ± 1.49 years.

- Sociodemographic and medical data.

The sociodemographic and medical data of the patients are summarized in  Table 1 and  Table 2 respectively. The sample was homogeneous because most of the variables present a non-statistically significant intergroup difference (*p*> 0.05).

**Table 1:**
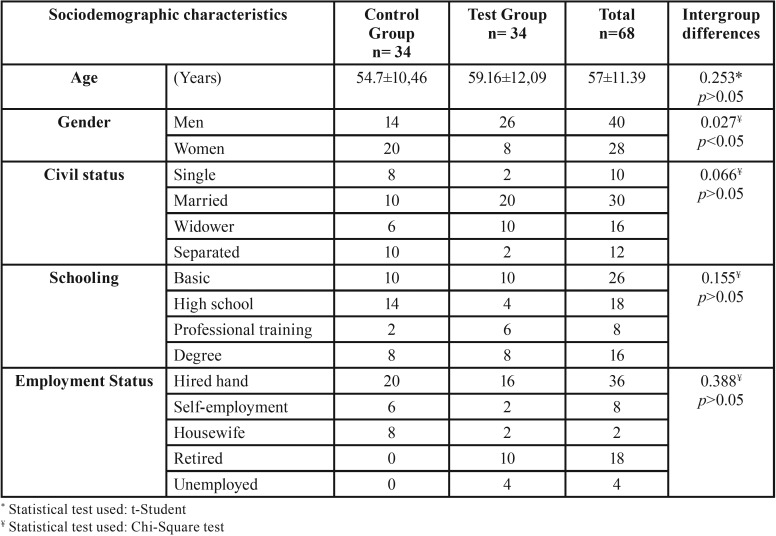
Sociodemographic characteristics of the sample.

**Table 2:**
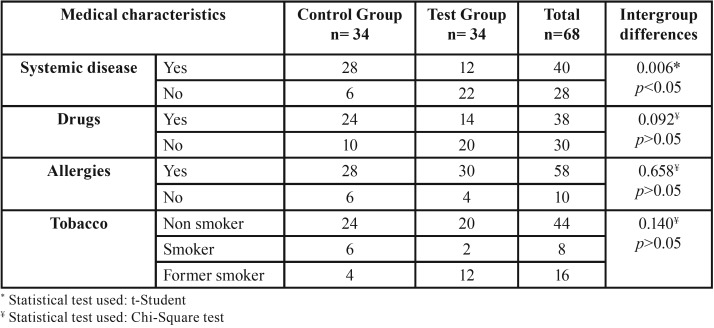
Medical characteristics of the sample.

- Results of the clinical parameters

•Plaque Index (PI)

The control group (CG) presented at baseline a mean plaque value of 0.676 ± 0.374, 0.588 ± 0.526 in the revaluation at 6 weeks and 0.509 ± 0.370 at 3 months. On the other hand, the test group (TG) presented at the baseline visit a value of 0.824 ± 0.541, 0.248 ± 0.3155 at 6 weeks and 0.480 ± 0.336 at 3 months. Statistically significant differences were observed at 95% (t-Student *p* = 0.041) between the two study groups at the time of the revaluation visit at 6 weeks ( Table 3).

**Table 3:**
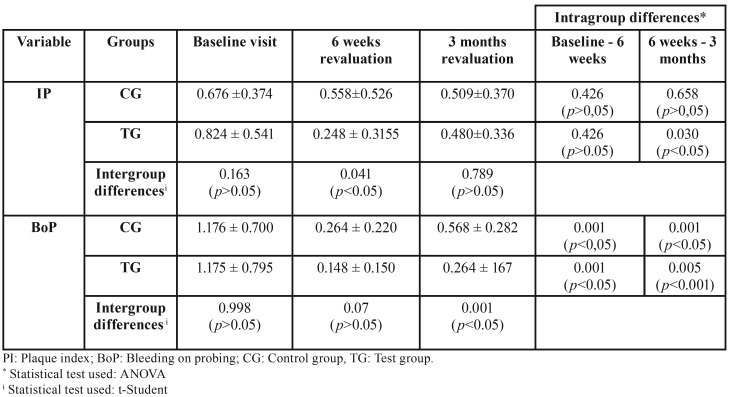
Plaque index and bleeding on probing variables during the study.

•Bleeding on Probing Depth (BoP)

**Figure 3:**
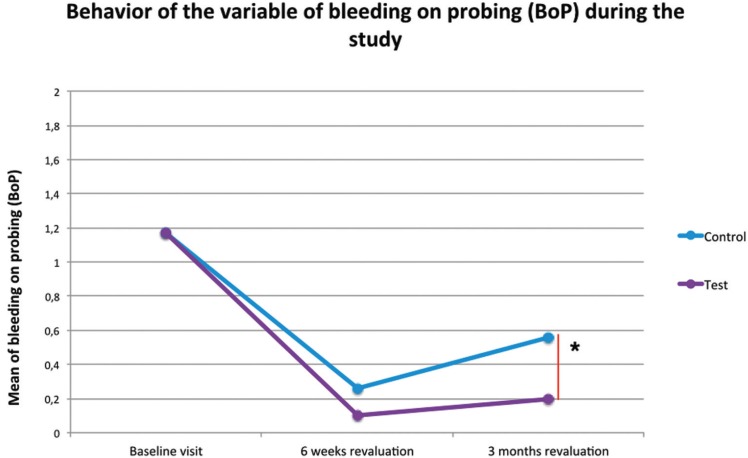
Behavior of the variable of bleeding on probing (BoP) during the study. Statistically significant difference (*p*<0.001). * Statistical test used: t-Student.

The GC presented an average baseline bleeding rate of 1.176 ± 0.700, 0.264 ± 0,220 at 6 weeks and 0.568 ± 0.282 at 3 months. On the other hand, the TG presented a value of 1.175 ± 0.795, 0.148 ± 0.150 at 6 weeks and 0.264 ± 167 at 3 months. Statistically significant differences were observed at 95% (t-Student *p* = 0.001) between the two study groups at the time of the 3-month revaluation visit ( Table 3). Figure 3 shows the behavior of this variable throughout the study.

•Probing pocket depth

The CG presented at baseline a mean probing pocket depth value of 1.303 ± 0.409mm, 1.137 ± 0.222mm in the revaluation at 6 weeks and 1.166 ± 0.263mm at 3 months. On the other hand, the TG presented at the baseline visit a value of 1.277 ± 0.347mm, 0.833 ± 0.374mm at 6 weeks and 1.068 ± 0.103mm at 3 months. Statistically significant differences were observed at 95% (t-Student *p* = 0.041) between the two study groups at the time of the revaluation visit at 6 weeks ( Table 4).

**Table 4:**
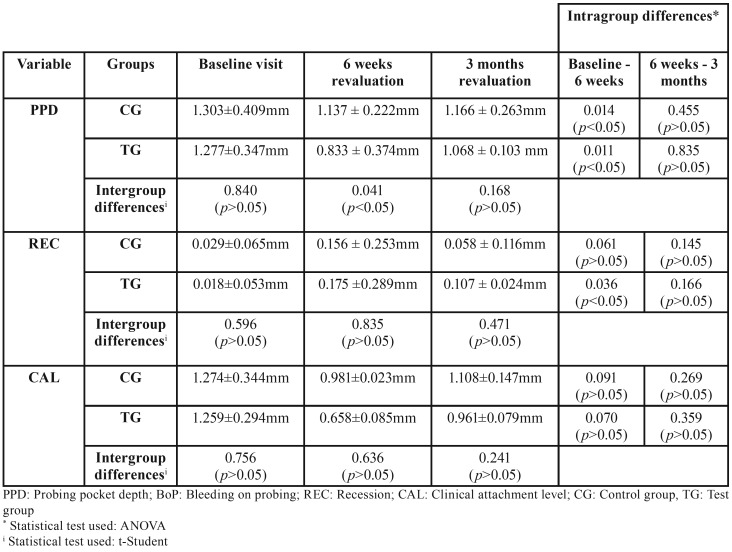
Probing pocket depth, recession and clinical attachment level variables during the study.

•Recession

The CG presented at baseline a mean recession value of 0.029 ± 0.065mm, at the 6-week revaluation 0.156 ± 0.253mm and 0.058 ± 0.116mm at 3 months. On the other hand, the TG presented at the baseline visit a value of 0.018 ± 0.053mm, 0.175 ± 0.289mm at 6 weeks and 0.107 ± 249mm at 3 months. There were no statistically significant differences at 95% in the behavior of this variable throughout the study ( Table 4).

## Discussion

The results of the present study show that those patients who underwent diode laser therapy as an adjunctive element to the conventional treatment of mucositis (test group), showed less bleeding at 3 months revaluation than those patients who only received conventional therapy (*p* <0.001). The control group also showed worse results in the rest of the clinical variables, however these differences were not statistically significant (*p*> 0.05).

- Plaque index 

Once the oral hygiene instructions were given, it was observed that in the reevaluation at 6 weeks the test group had shown more improvement in the reduction of the plaque index (*p* <0.05). However, at 3 months both study groups returned to present similar biofilm values. These results agree with those obtained by the Aimetti *et al.* in 2019, which shows an improvement in the plaque index for both groups significantly (*p* <0.001) but without differences between them at the 3-month revaluation (14).

The fact that both study groups improve plaque index values is mainly due to oral hygiene instructions offered by researchers to patients. The decrease at 6 weeks seems to be due to the bio-stimulation effect of the diode laser, which by facilitating the formation of epithelial sealing reduces plaque retention. However, patients tend to demotivate over time, which results in a greater accumulation of biofilm in the revaluation at 3 months (14).

- Bleeding on probing 

In our study, both groups improve significantly in bleeding on probing values (*p* <0.001), obtaining statistical significance between both groups in the revaluation at 3 months (*p* <0.001). Other studies such as Lerario *et al.* and Al-Amri *et al.* also obtained statistically significant differences (*p* <0.05) in the decrease in bleeding at probing between both study groups (13,15), these studies were those with the longest follow-up time (12 months). On the other hand, studies with shorter follow-up times do not observe differences in bleeding at probing between both study groups, despite having statistically significant differences in plaque index. In our study, the difference between bleeding at probing in the test group and the control group was increasing according to the follow-up time, regardless of the increase in plaque index.

Aoki *et al.* reported in their review that several types of lasers, including diode laser, have bactericidal properties. Due to their photothermal effect, the bacteria are evaporated, destroyed or denatured by the effect of the laser, which results in their devitalization or inactivation (16,17). These bactericidal properties together with biostimulation mechanism reduce the inflammation of the peri-implant tissues (18,19).

- Probing pocket depth

Both groups improved in their probing pocket depth measurements (*p* <0.05). However, the test group showed greater improvement than the control group, being this difference at the 6-week revaluation statistically significant (*p* <0.05). Nevertheless, at 3-month revaluation the measurements increased slightly, obtaining better results for the test group but without statistical differences. Authors such as Al-Amri *et al.* and Aimetti *et al.* obtained similar results in their studies (14,15). In these studies, as in ours, plaque indexes were balanced between both study groups, this increase in plaque index makes epithelial seal formation difficult, causing, despite the effect of the laser, slight changes in the probing pocket depth.

- Recession

The peri-implant mucosal recession increased slightly in both study groups, but without statistically significant differences at any time during the study (*p* <0.05). Previous studies such as Deppe *et al.* or Thierbach *et al.* also reported increases in mucosal recession after the application of the diode laser (20,21). When the inflammation decreases, so does the edema of the tissues and with a smaller volume the true loss of mucosal insertion is evidenced (22).

Various limitations must be taken into account in this study. The published scientific literature on the topic is very limited. In addition, the studies differ in the definition of peri-implant mucositis, the non-surgical treatment applied to the control group and the diode laser protocol. All this makes it difficult to compare our results with those obtained in the rest of the studies. Finally, only clinical variables were taken into account, more studies are needed that approach the issue from a microbiological and immunological point of view, in order to be able to evaluate the true potential of the diode laser in the treatment of peri-implant pathologies.

Therefore, with the limitations of this study, the hypothesis that the diode laser can bring additional clinical benefits to conventional non-surgical therapy of peri-implant mucositis is accepted. A better response of the gingival index was obtained, especially in bleeding on probing, which avoids a significant decrease of the inflammation in the peri-implant tissues, showing a new line of research in the application of new technologies in the treatment of the peri-implant pathologies.
